# Age-Friendly Cities and Communities: State of the Art and Future Perspectives

**DOI:** 10.3390/ijerph18041644

**Published:** 2021-02-09

**Authors:** Joost van Hoof, Hannah R. Marston

**Affiliations:** 1 Faculty of Social Work & Education, The Hague University of Applied Sciences, 2521 EN Den Haag, The Netherlands; 2 Faculty of Environmental Engineering and Geodesy, Institute of Spatial Management, Wrocław University of Environmental and Life Sciences, 50-357 Wrocław, Poland; 3 Health & Wellbeing Strategic Research Area, School of Health, Wellbeing & Social Care, The Open University, Buckinghamshire MK7 6HH, UK; hannah.marston@open.ac.uk

The number of older adults is increasing rapidly, and this demographic shift places an increased level of strain and tension on the various international healthcare and welfare systems. The vast majority of older adults wish to age in place. Many make use of long-term care services, including homecare, rehabilitation services, and social support, as well as home modifications and technology, although, contrary to popular belief, this is not the majority of older people. One way to support older people to live the lives they wish to live is through the Age-Friendly Cities and Communities initiative, a world-wide programme launched by the World Health Organization (WHO) in 2007 [1] in order to make cities more tuned to the needs and requirements of older citizens [2,3,4]. The World Health Organization defines Age-Friendly Cities and Communities as follows: “*In an age-friendly community, policies, services and structures related to the physical and social environment are designed to support and enable older people to “age actively”—that is, to live in security, enjoy good health and continue to participate fully in society*” [5].

The WHO published an age-friendly cities guideline that was accompanied by a checklist of essential features of age-friendly cities. This checklist was based on the results of the WHO Global Age-Friendly Cities project consultation in 33 cities in 22 countries [6]. For the checklist to be effective, older people must be involved as full partners. In assessing a city’s strengths and deficiencies, older people describe how the checklist of features matches their own experience of the city’s positive characteristics and barriers. They should play a role in suggesting changes and in implementing and monitoring improvements [1].

In the second decade of the WHO programme, it is fair to say that it contributed to the emancipation of older people, namely that their voices were heard in urban governance and planning, as well as in the programming of services for older persons all over the world. Yet, there are many unanswered questions and challenges lying ahead. For instance, how is the complex interplay of needs and demands of older persons [7,8,9] included into the design of age-friendly solutions in every possible domain? In order to create age-appropriate living environments, it is of the utmost importance to involve older people in the design of their living environment, particularly because the importance given to neighbourhoods in old age can vary greatly [10]. Another question is what the age-friendly agenda and its recommendations mean for older people living in such age-friendly cities and communities. In short, what do citizens notice in their everyday lives of the efforts to be or become an age-friendly city? Additionally, how can you really tell that a city is age-friendly, for instance, by measuring the age-friendliness of cities using core indicators [11,12,13,14,15,16,17], and that being part of this global network of cities is not just a tokenistic attempt of urban governments to show a friendly image to the outside world? Do age-friendly cities and communities really offer better living conditions and environments to their older citizens and the overall population than non-age-friendly cities? In short, what does it truly mean to be age-friendly in practice? Relevant for various stakeholders is the question whether the programme is still up to date after being in use for nearly a decade and a half.

The WHO published a report in 2018 [18] with the subtitle “*Looking back over the last decade, looking forward to the next*”, in which technology is explicitly mentioned as a support for age-friendly environments. In 2019, Marston and van Hoof [19] presented a critique of the WHO’s Age-Friendly Cities and Communities model, as *technology is not explicitly considered in this model. Their paper discusses the gaps in the WHO’s framework in the field of technology and provides insights and recommendations for expansion of the model for application in the context of countries with a high human development index (HDI) that wish to be fully age-friendly. The question was raised if* the age-friendly programme prepares cities to be truly age-friendly in a world that is increasingly moving towards being a digital or even smart society? How considerate is the age-friendly movement of the inclusion of digital technologies, embracing their potential to the fullest? *Over the decades, technology has become essential for contemporary and future societies, and even more imperative as the decades move on.* Podgórniak-Krzykacz et al. [20] also called for smart cities to seek to ensure meeting the needs of older citizens and promoting solutions tailored to their digital literacy, digital skills, and perception capabilities.

The world of the 2020s needs answers to the abovementioned questions and challenges. These questions, therefore, provide some of the rationales for the Feature Paper Special Issue entitled “*Age-Friendly Cities and Communities: State of the Art and Future Perspectives*” which is published in the section of Health Care Sciences and Services of the International Journal of Environmental Research and Public Health (IJERPH).

The primary focus of this Feature Paper Special Issue is to critically assess the state-of-the-art Age-Friendly Cities and Communities programme. It adds to a previous special issue by van Hoof et al. [21] entitled “Creating age-friendly communities: Housing and technology” of MDPI’s Healthcare in the following manner, by providing a wider scope of papers that provides a more diverse set of recommendations for practice and future work. The purpose of this Feature Paper Special Issue was to publish high-quality research papers, commentaries, and review articles addressing recent advances in age-friendly cities. There are eight domains of an age-friendly city, specifically social participation, communication and information, civic participation and employment, housing, transportation, community support and health services, outdoor spaces and buildings, and respect and social inclusion. In addition, this Feature Paper Special Issue also considered the importance of (geron)technology and digital solutions in relation to age-friendly environments.

For this Feature Paper Special Issue entitled “*Age-Friendly Cities and Communities: State of the Art and Future Perspectives*”, a total of 29 papers [22,23,24,25,26,27,28,29,30,31,32,33,34,35,36,37,38,39,40,41,42,43,44,45,46,47,48,49,50] were recently published on different topics related to this subject matter. Of the published papers, seven papers [22,23,24,25,26,27,28] related to age-friendly neighbourhoods, cities, communities and societies, three papers [29,30,31] explored innovative approaches to housing, two papers [32,33] concentrated on age-friendly transportation, four papers [34,35,36,37] focused on innovative practices in the domain of cure and/or care for older citizens, four papers [38,39,40,41] related to respect and social inclusion, and nine papers [42,43,44,45,46,47,48,49,50] dealt with the consideration of technology in an age-friendly city or community.

*Age-friendly neighbourhoods, cities, communities and societies*

The seven papers in this section provide a wide range of insights, which add to the current scientific base in the domain of urban ageing [4,51,52,53,54,55,56,57,58,59]. The role of neighbourhoods is studied through various methodological approaches. In addition, new approaches to evaluating age-friendliness of a city or community are presented, as well as directions for future research policy and practice.

The paper by Versey et al. [22] from the United States of America explored neighbourhoods within age-friendly cities and communities and their role in shaping the everyday lives of older adults. The study explored the impact of gentrification on older adults and explored indirect displacement due to the change in character and social identity of a neighbourhood, which is one of the consequences of gentrification. The perceptions of older people concerning indirect displacement were studied in New York City and were characterised by perceived cultural shifts and housing concerns among adults. The implications of indirect displacement are potential threats to ageing-in-place in age-friendly cities.

Von Faber et al. [23] presented a study on participatory video design as an empowering approach to collect experiences and perceptions of older people focusing on the age friendliness of their city or neighbourhood. They described how this co-creation method can add to specific knowledge about the needs and wishes of older people about the improvement and/or preservation of their environment. Older participants produced short films on the topics that were perceived as important from their own perspective regarding their neighbourhood. Topics of the films included communication and information, outdoor spaces, social relations, and community support.

Sterns et al. [24] presented a survey from the city of Akron in the state of Ohio. In order to provide direction for future ageing initiatives, an assessment of Akron current state was conducted in early 2020. A total of 656 individuals participated and rated Akron from good to excellent. Most Akronites like and use their neighbourhood parks, find their streets well-lit, and feel safe walking in their neighbourhood. Conversely, more than 80% of respondents indicated how they disagree with the notion of them being disconnected from the community. Overall, Akron benefitted from its historical efforts to become age-friendly.

The study by Davern et al. [25] set out with a major critique of the age-friendly community movement, which argued for a more clearly defined scope of actions, the need to measure or quantify results and increase the connections to policy and funding levers. The scholars provided a quantifiable spatial indicators framework to assess local lived environments according to each of the eight domains of the WHO. The selection of the spatial indicators can be applied within local neighbourhoods, census tracts, suburbs, municipalities, or cities with minimal resource requirements other than applied spatial analysis.

Dikken et al. [26] also stressed the need for validated instruments to assess the age-friendliness of cities and communities. They developed and validated a questionnaire measuring age-friendliness, providing full transparency and reproducibility, coined the Age Friendly Cities and Communities Questionnaire (AFCCQ). Their process of development and validation resulted in a valid, psychometrically sound, comprehensive, 23-item questionnaire (Figure 1). Only those people aged 65-years or over (with an exception of 10 people aged between 60 and 65 from an existing database, who identified as older citizens) who lived in their own home were included. The AFCCQ can be used to measure older people’s experiences regarding the eight domains of the WHO Age-Friendly Cities model and an additional financial domain. The AFCCQ allows practitioners and researchers to capture the age-friendliness of a city or community in a numerical fashion, which helps monitor the age-friendliness and the potential impact of policies or social programmes.

A commentary by Marston et al. [27] described and presented the existing Blue Zones^®^ checklists and set out initial thoughts and explorations relating to the checklists. Additionally, Marston and colleagues discussed the two age-friendly frameworks by the WHO [1] as well as by Marston and van Hoof [19], and discussed the current gaps associated to the current Blue Zones^®^ checklists. This commentary presented a series of recommendations for a roadmap to be considered by scholars, in conjunction with various industrial and third sector actors, to consider alternative and innovative approaches moving into the 21st century.

Rémillard-Boilard et al. [28] focused on driving the ‘age-friendly’ agenda, notably through the WHO’s Global Network of Age-Friendly Cities and Communities. Little is known about the progress made by cities developing this work around the world. Therefore, their work addresses this research gap by comparing the experience of eleven cities located in eleven countries. Using a multiple case study approach, the authors explored the key goals, achievements, and challenges faced by local age-friendly programmes. They identified four priorities the age-friendly movement should consider to expand its development: (1) changing the perception of older age; (2) involving key actors in age-friendly efforts; (3) responding to the (diverse) needs of older people; and (4) improving the planning and delivery of age-friendly programmes. These conclusions carry implications for both research and policy in the field of age-friendly cities and communities.

*Innovative approaches to age-friendly housing*

The three papers in this section provide an additional knowledge base to the wider body of knowledge that exists in the field of age-friendly housing and ageing-in-place [60,61,62,63,64,65,66].

The paper by Rusinovic et al. [29] built on a previous contribution [67], which concerned the qualitative investigation of co-housing communities for older people in The Netherlands. Such communities offer an in-between solution for older people who do not want to live in an institutional setting but prefer the company of their age peers. Rusinovic et al.’s study focused on housing initiatives that offer innovative and alternative forms of independent living which deviate from mainstream housing arrangements. The study investigated how the founders dealt with challenges of establishing and governing such ‘rebellious’ innovative living arrangements for older people in the highly regulated context of housing and care in The Netherlands. Qualitative, in-depth interviews with social entrepreneurs, directors, and supervisory board members were conducted. These founders encountered various obstacles which are often related to governmental and sectoral rules and regulations. Their stories about successes and failures demonstrate dthe opportunities and constraints of innovative entrepreneurship at the intersection of housing and care.

The study by Bennetts et al. [30] dealt with thermal comfort in the homes of older people and is part of a larger project *Improving the thermal environment of housing for older Australians* [68,69]. This paper described the fundamental approach for developing the guidelines, using data from the study participants and the concept of personas to develop a total of six discrete ‘thermal personalities’. The thermal personalities represented different approaches to achieving thermal comfort, considering a wide range of factors including personal characteristics, ideas, beliefs and knowledge, house type, and location. Basing the guidelines on thermal personalities highlights the heterogeneity of older people and the context-dependent nature of thermal comfort in the home, making the guidelines more user-friendly and useful.

Sengers and Peine [31] presented an overview of pilot projects in the field of housing, which are referred to as ‘sociotechnical experiments’. These experiments embody different kinds of promising futures and provide direction to current developments in the emerging domain of age-friendly homes. The authors provided an overview of 53 ongoing sociotechnical experiments from The Netherlands, France, Ireland, and Poland. Most of the innovations tested in these experiments were not primarily material or technical, but primarily social or conceptual in character, and there were seven distinct innovation pathways in the experiments.

*Age-friendly transportation*

The following two papers deal with age-friendly transportation, which is becoming increasingly important in the light of smart mobility. At the same time, classic indicators for the quality of transportation, such as affordability, availability, and accessibility, remain important.

The study from Canada by Klicnik and Dogra [32] looked at the active transportation facet as an affordable and accessible form of transportation that facilitates the mobility of older adults in their communities. Age-friendly cities often do not adequately address active transportation. The study set out to identify and understand the constraints to active transportation that older adults experience to inform the development of viable solutions. The study conducted focus groups with community-dwelling older adults. Themes pertaining to environmental, individual, and task constraints, as well as their interactions, were identified. The study showed that constraints to active transportation interact to exacerbate one another, and that there is an opportunity to minimise such constraints by implementing age-friendly policies and practices.

Loos et al. [33] explored older people’s (smart) mobility, with a particular interest in public transport, considering digital elements through a narrative literature review. Their study aimed to conceptualise transport as a core element of a smart, age-friendly ecosystem, and to propose a justice-informed perspective for the study of age-friendly smart mobility. Their findings contribute towards a framework for the evaluation of age-friendly smart transport that comprises mobility practices, digital data, digital networks, material/physical geographies and digital devices and access. The authors coined the term ‘mobility digital ecosystem’ to describe this framework, which comprises mobility practices, digital data, digital networks, material geographies, digital devices, and access to services.

*Innovative practices in age-friendly cure and care*

The following four papers dealt with innovative practices in age-friendly cure and care, whether it concerns hospital care services, older people’s health information needs, the innovation of long-term care services, or models of care. All papers dealt with evidence-based or evidence-informed approaches to practical innovations [7,8].

The study by Ferrari et al. [34] from Italy focused on age-friendly hospital care. Consultation–Liaison Psychiatry Services (CLPS) are significantly involved in providing age-friendly hospital care. Such services perform psychiatric assessment for older people who are hospitalised with suspected medical–psychiatric comorbidities and support ward teams in a biopsychosocial-oriented care management. Changes in features of the population referred to a CLPS over a 20-year course were analysed and discussed, especially comparing older and younger referred subjects. The number of referrals for older patients significantly increased over the past 20 years. The analysis outlined recurring patterns that should guide future clinical, training, and research activities.

A study from the Russian Federation by Ziganshina et al. [35] presented the case of Kazan, the capital of Tatarstan, as a potential age-friendly city. This survey study assessed health information needs of the ageing population and the challenges these older people face in improving their health and longevity. Older people (60+ years) were less positive about their quality of life, who more often took medication on a daily basis, who also encountered age-related health problems and rated their overall quality of life as unsatisfactory. Awareness in evidence-based approaches was higher within health professions, and health information needs did not differ between age or gender groups or people with satisfactory and unsatisfactory quality of life. The minority (10%) were aware of ageism without age or gender differences. The low awareness calls for the need of interventions for both care recipients and professionals in order to move the age-friendly agenda forward.

Luijkx et al. [36] dealt with long-term care organisations for older adults that are expected to provide person-centered care in the complex arena of The Netherlands. In order to address the challenges of the innovative Dutch context, these organisations increasingly use scientific knowledge to evaluate and innovate long-term care. Their paper described how co-creation is a key factor in the success of improving long-term care for older adults, and how scientific knowledge is created by working together with partner organisations and how societal impact is achieved.

De Boer et al. [37] presented the case of alternative care environments for regular nursing homes. Insight is lacking on how to translate evidence-based knowledge from theory into a congruent care model in regular practice. This study reported on the co-creation and redesign of an alternative nursing home model in The Netherlands. A participatory research approach was used to co-create ‘the Homestead care model’ with researchers, practitioners, and older people, following an iterative process. Achieving positive outcomes for people with dementia, (in)formal carers, and the community is dependent on how well the physical, social, and organisational environments are congruently designed.

*Respect and social inclusion in an age-friendly city*

The following four papers dealt with the social environment(s) of age-friendly cities and communities, and the inclusion, representation, and participation of older people, as well as the role gender plays in the perception of age-friendliness.

Ronzi et al. [38] focused on the social environment of the age-friendly city’s model. Using a photovoice methodology within a community-based participatory research approach, their study drew on photographs, interviews, and focus groups among older residents (60+ years) living in Liverpool to explore how the city promotes respect and social inclusion. Their study provided novel insights into how (i) respect and social inclusion are shaped by aspects of both physical and social environment, and (ii) the accessibility, affordability, and sociability of physical spaces and wider social processes (for instance, neighbourhood fragmentation) contributed to or hindered participants’ health, well-being, intergenerational relationships, and feelings of inclusion and connection. Their findings suggested that respect and social inclusion are core to an age-friendly city, and relevant across all eight domains.

Codd [39] presented an interdisciplinary article, bringing together perspectives from gerontology, criminology, penology, and social policy to explore the research, policy, and practice on age-friendly cities and communities for people who are ageing within prison settings across many countries. There is a general omission of consideration of the place of the prison and prisoners within the broader context of age-friendly cities and communities. Codd identifies the potential for integration and for cross-disciplinary research in this context, concluding with recommendations for developing inclusive research, policies, and evaluation frameworks which recognise and include prisons and older prisoners, both during and after incarceration.

Blakey and Clews [40] presented a study from Tāmaki Makaurau Auckland in Aotearoa New Zealand, which houses the largest Polynesian population of any global city. This case study inquiry applied the bricolage methodology to provide diverse contextual perspectives of this unique Polynesian setting, prior to exploring interview narratives of three Auckland Council’s Seniors Advisory Panel members. Service-learning recommendations included co-developing a sustainable co-governance framework for an independent Steering Group to enable empowered active ageing for all residents, and a succession plan enabling the timely transfer of knowledge and skills to empower incoming Auckland Council’s Seniors Advisory Panel members.

The study by del Barrio et al. [41] analysed the interaction between age-friendliness (physical and social) and subjective well-being through a survey among people aged 55-years and over in the Basque Country in Spain. The predictive power of age-friendliness over subjective well-being was analysed using linear regression models separated by sex. Among the predictors of well-being in men, the coexistence stood out as a safety and support network. In women, the neighbourhood proved to be a very important resource. The findings may contribute to interventions promoting more effective strategies that enhance older people well-being from a gender perspective.

*Technology and the age-friendly city*

The following nine papers dealt with technological solutions and ageing built on the foundations laid out by Marston and van Hoof [19], who laid down the importance of technology and digitalisation as a third pillar for age-friendly cities and communities. Aspects of use-friendly and sustainable design, technology acceptance, and aspects of implementation and needs of carers were all acknowledged in the following contributions [8,9,70,71,72,73,74,75,76,77].

The study by Baraković et al. [42] was a deliverable of COST Action CA16226 ‘Indoor living space improvement: Smart Habitat for the Elderly’ and presents the collaborative efforts of researchers from Europe and North America. This review focused on the quality of life through the concept of personalised ageing. Information and communication technologies (ICT) are subject to constant and rapid development and can contribute to the goal of an improved quality of life for older adults. The systematic review of the state-of-the-art literature and patents in this field was based on a framework for the quality of life of older adults. Selected ICT solutions covered by articles and patents were intended for older adults and were validated by them. The study presented several recommendations that can help move the agenda concerning the quality of life of older people and personalised ageing with the use of ICT solutions forward. This paper was related to a comprehensive and structured analysis of the existing literature in the field of the Web of Things, and the user’s quality of experience towards used technology [78].

The study by Anghel et al. [43] from Romania set out with the limitations posed by a decreasing workforce in the supply of care and social services. The development of smart, physical, social, and age-friendly environments was part of the solution. The authors conducted a survey of smart environments and robot assistive technologies that offer support for the independent living and providing age-friendly care services. Two cases were presented of services that are innovatively using assistive technologies for the assessment and delivery of timely interventions for polypharmacy management and for social and cognitive activity support in older adults. The study also provided a top-level architectural view of these services focusing, on details about technology usage, end-user interaction flows, and data models.

The study by Liddle et al. [44] from the United Kingdom focused on social connectedness in later life. The authors explored the design opportunities and role of technology for connectedness within a geographically local community context through interviews with older people and a linked ideation workshop. Shared concerns and negative perceptions around local relationships, connections, and characteristics of the geographical area were identified. Local connectedness through technology was largely absent from day-to-day life and even perceived as contributing to disconnection. A thoughtful consideration of the role of technology in optimising social connections within age-friendly communities is needed.

Silvius et al. [45] presented results on the use and acceptance of commercially available technology by home dwelling older citizens. A comparison was made between self-efficacy and perceived physical and mental quality-of-life-related parameters on an intervention location of 279 households and a control location of 301 households located in The Hague in The Netherlands. Technology adoption was significantly associated with perceived physical quality of life, depending on the number of technology interventions used. A higher number of adopted technologies was associated with a stronger effect. The study showed that successful and effective adoption of technology by older people is feasible with commercially available products amongst home dwelling older citizens.

In their study on smart and age-friendly cities in Romania, Ivan et al. [46] compiled an overview of public policy and practice. Smart cities are one of the technological-driven initiatives that may help create an age-friendly city. Few research studies analysed emerging countries in terms of their national strategies associated to smart or age-friendly cities. Through document analysis, current initiatives at the local, regional, and national level addressing the issue of smart and age-friendly cities in Romania were investigated. To date, Romanian smart home initiatives have limited connection to the age-friendly cities agenda.

Freeman et al. [47] studied the intergenerational effects on the impacts of technology use in later life through an online survey. As the use of technology becomes an integral part of daily life for all persons, including older adults, it is important to investigate how the perceptions and use of technology intersect with intergenerational relationships. Descriptive and thematic analyses suggest that older adults are not technologically adverse and leverage intergenerational relationships with family and friends to adjust to new technologies and to remain connected to adult children and grandchildren, especially when there is a large geographical separation between them. The intergenerational support to adopt to new technologies has important implications to support older persons to remain independent and to age-in-place, in both age-friendly cities and in rural geographies.

Pedell et al. [48] presented two case studies, one focusing on older adults using activity wearables for health self-management in the neighbourhood, and one focusing on older adults engaged in social prescribing activities in the community. A co-design and citizen-based approach was applied. Results suggested how the convergence of the often-siloed age-friendly city components based on older adults’ goals and input can lead to better social participation and longer-term health outcomes. The authors proposed that the digital, physical, and social aspects need to be considered in all domains of age-friendly cities to achieve benefits for older adults.

Marston et al. [49] presented a theoretical case study to explore how digital technology has played an integral role during COVID-19, assisting various sectors of the community and demonstrating that smart cities can provide opportunities to respond to many future societal challenges. Although we need to create future smart age-friendly ecosystems to meet these needs, technology still does not feature in the WHO eight domains of an age-friendly city. This paper expands upon Marston and van Hoof’s [19] ‘Smart Age-friendly Ecosystem’ (SAfE) framework, and explores how digital technology, design hacking, and research approaches can be used to understand a smart age-friendly ecosystem in a postpandemic society. By exploring a series of case studies and using real-life scenarios from the standpoint of COVID-19, the authors proposed the ‘Concept of Age-friendly Smart Ecologies (CASE)’ framework.

Reuter et al. [50] set out by stating that the WHO’s age-friendly city initiative emerged as a response to the intersecting global trends of population ageing and urbanisation. A third global trend—digitalisation—is largely overlooked. The authors explored older adults’ digital citizenship in an age-friendly city in the north of England through interviewing, observations, and field notes from design workshops as part of an ongoing participatory action research project. The analyses focused on two age-friendly domains, namely communication and information and civic participation. The authors saw the need to reframe the role of digital technologies within the age-friendly city, broadening the scope from accessibility towards enhancing digital citizenship opportunities.

In this special issue, a rich palette of views and studies was presented. After taking notice of this vast and diverse body of knowledge, the question emerges of how to move forward from here. How can this knowledge contribute to the further development of age-friendly cities and communities which benefits people of all ages?

We would like to call upon the wider scientific community, local, regional, and national governments, social enterprises, local and national businesses (such as construction companies, tradesmen etc.), and industry leaders (such as design agencies, manufacturing, fin-tech etc.), architects and urban planners, construction companies, and the creative industries, which can afford citizens of all ages various opportunities for active engagement in various elements of their respective age-friendly cities and ecosystems.

As Wetle ([79], p. 1930), posited, “designing and implementing age-friendly communities, health systems, or ecosystems requires long-term commitment and considerable resources, which necessitates a strong and effective champion who can bring together potential partners, share a compelling vision, and provide energy and leadership to the continuing effort”. Therefore, it is laudable that an inclusive and age-friendly society is becoming more mainstream in Western societies through actions of the media. By working together between the disciplines and creating truly multisectoral actions, a genuine age-friendly society may be achieved for current and future generations. Such actions may even go beyond the current borders of the age-friendly movement, covering age-friendly public health and health systems, age-friendly states, and age-friendly universities [80].

The sharing of metrics and outcomes is one of the essential keys for successful action. This Feature Paper Special Issue entitled “*Age-Friendly Cities and Communities: State of the Art and Future Perspectives*” is one of the ways to expand and disseminate the knowledge on age-friendly cities and communities and to facilitate the actual age-friendliness of cities and communities agenda and narrative further. The body of knowledge presented here in this special issue acknowledges the importance of the interplay surrounding ageing, urbanisation, and digitalisation (technology). Additionally, this special issue affords scholars, stakeholders, regional and national policymakers and governments, and various actors within industry to understand, learn, and act upon to ensure the interconnected and adjoining facets associated to the quality of life for both younger and older people are met. Furthermore, this information and knowledge can and should benefit members of the wider communities (for instance, younger generations, people with chronic health/life-limiting conditions etc.) in our respective societies through real actions, instead of holding a mere promise for age-friendliness through the endless development of more tokenistic and policy-oriented age-friendly agendas, models, and frameworks that are based on outdated references from the previous century.

## Figures and Tables

**Figure 1:**
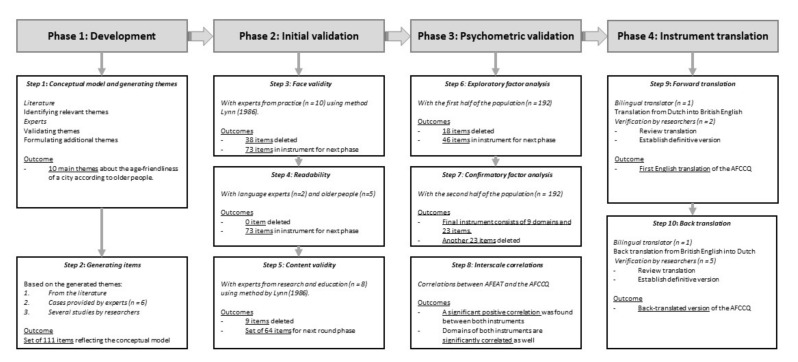
Flowchart representing the phases and steps for developing the Age-Friendly Cities and Communities Questionnaire (AFCCQ). Step 7 had 9 domains and 23 items as the final outcome. Please note that in §2.2.2. of the paper [26] step 2.1 should be step 3, and in §2.2.4 of the paper, step 2.3 should be step 5.
